# An Energy-Efficient MAC Protocol Using Dynamic Queue Management for Delay-Tolerant Mobile Sensor Networks

**DOI:** 10.3390/s110201847

**Published:** 2011-02-01

**Authors:** Jie Li, Qiyue Li, Yugui Qu, Baohua Zhao

**Affiliations:** 1 Department of Electronic Engineering and Information Science, University of Science and Technology of China, Hefei, Anhui 230027, China; E-Mails: myl@mail.ustc.edu.cn (J.L.); cnygqu@ustc.edu.cn (Y.Q.); 2 Department of Computer Science and Technology, University of Science and Technology of China, Hefei, Anhui 230027, China; E-Mail: bhzhao@ustc.edu.cn (B.Z.)

**Keywords:** delay-tolerant mobile sensor networks, minimal probe frame, Wait to Send, Ready to Receive, queue management, priority, medium access control

## Abstract

Conventional MAC protocols for wireless sensor network perform poorly when faced with a delay-tolerant mobile network environment. Characterized by a highly dynamic and sparse topology, poor network connectivity as well as data delay-tolerance, delay-tolerant mobile sensor networks exacerbate the severe power constraints and memory limitations of nodes. This paper proposes an energy-efficient MAC protocol using dynamic queue management (EQ-MAC) for power saving and data queue management. Via data transfers initiated by the target sink and the use of a dynamic queue management strategy based on priority, EQ-MAC effectively avoids untargeted transfers, increases the chance of successful data transmission, and makes useful data reach the target terminal in a timely manner. Experimental results show that EQ-MAC has high energy efficiency in comparison with a conventional MAC protocol. It also achieves a 46% decrease in packet drop probability, 79% increase in system throughput, and 25% decrease in mean packet delay.

## 1. Introduction

At present, most wireless sensor network (WSN) applications, *i.e.*, networks composed of large numbers of distributed sensor nodes that sample data, are limited to static sensor nodes. Such networks are good for natural environment monitoring and medical treatment, but their mechanisms are not sufficient for sensor applications [1,2] of sparse (low node density) networks that may appear in the future. In particular, they cannot deal with sensor node mobility. Motivated by the research on networks with low and intermittent connectivity, reference [3] proposes a delay-tolerant mobile sensor network (DTMSN) of wearable nodes that move with their carriers, gather information, and deliver it to a sink. The DTMSN distinguishes itself from conventional WSNs by its node mobility, sparse connectivity, delay tolerability [4–6] and highly dynamic network topology. Its sparse node density results in poor network connectivity [7]. Moreover, DTMSN possesses high delay tolerance and is affected by changes in queue size. These characteristics pose the following challenges for the design of MAC protocols.

- Power constraints: energy efficiency is often a major concern in WSNs due to the nodes’ limited batteries. DTMSN sensor nodes must be conveniently portable. That means they need to have a most small button battery, a very limited energy source. Furthermore, the dynamic network topology reduces data transfer success rate, which in turn increases energy consumption. Therefore, an effective protocol mechanism to make efficient use of the limited energy of the nodes and thus extend the lifetime of the network is essential.
- Data delivery scheme: because of the sparse node density and high dynamic network topology of DTMSNs, mobile sensors are intermittently connected. This calls for the utmost use of the temporarily available communication links [3]. The data delivery scheme is the key to getting an adequate data transfer success rate.
- Queue-management strategy: in a DTMSN, data messages are delay-tolerant so that there is always a data queue in the memory of each sensor node ready for packet transmission. Hence, an appropriate queue management scheme is needed to sort the data messages in the queue, to determine which data message is to be sent when the sensor meets others or which message is to be dropped when the queue is full. Such a scheme should be able to avoid untargeted transfers, reduce packet transmission failures and meaningless retransmissions, and increase energy efficiency.

To reduce energy consumption, S-MAC [8] uses the RTS-CTS mechanism to wake up the neighbors of the sender and receiver after the packet is delivered; it avoids schedule misses and halves latency. T-MAC [9] improves on the design of S-MAC by shortening the period awake if the channel is idle. B-MAC [10] uses preamble sampling and eliminates synchronization to reduce energy consumption. X-MAC [11] and AS-MAC [12] maintain low power communications by using a shortened preamble approach. W-MAC [13] and CI-MAC [14] combine the MAC and routing functionalities to minimize the sleep delay and conserve energy in large-scale WSNs. The protocols above focusing on energy saving are usually used in WSNs. However, the network topology changes frequently in a mobile environment, and the data transmission fails if the topology information updates untimely. [15] put forward a dynamic queue management way to deal with the incoming data packets but did not bring an optimal solution to achieve energy saving. Thus these MAC protocols do not work well in DTMSNs. To overcome their limitations, this paper proposes an energy-efficient MAC protocol using dynamic queue management, dubbed EQ-MAC. EQ-MAC uses a minimal probe frame (MPF) to complete neighbor discovery and updating and has a transfer mechanism initiated by the target receiver to valid successful data transmission and an effective data queue management strategy to deal with priority queuing and manage the data in the queue. MPF implements the neighbor detection with the shortest frame. Thanks to the MPF, the neighborhood table maintained by each node can be updated timely, and the node which is ready to send the data packet can detect the sink node as soon as possible. Each node sends an MPF periodically. A node that receives the MPF will update its neighbor table and check whether it has data to send to the target neighbor. If it has, it rapidly sends a WTS (Wait To Send) as a response to tell the sink the time needed to complete the data packet transfer and to tell other neighbors to go to sleep and save energy until the transfer finishes; then as a sender, it starts to transmit the data packet to the target node after receiving a RTR (Ready To Receive) from the sink. Otherwise, the node goes to sleep until the next waking period arrives. If a sensor node has a data queue waiting for sending, it prioritizes the dynamic queue data as either high-priority packets (H-Pkt) or low-priority packets (L-Pkt). If it meets two target sinks, it will process the H-Pkts before the L-Pkts.

The rest of the paper is organized as follows: Section 2 describes the design of the EQ-MAC protocol. Section 3 introduces the queuing model to analyze the protocol, and Section 4 describes the optimization for the following experiments. Typical performance results validated through the experiments are shown in Section 5. Section 6 presents the conclusions.

## 2. Design of EQ-MAC Protocol

EQ-MAC uses MPF to update the neighbor table so as to validate communications among sensor nodes in order to deal with poor network connectivity. To deal with the DTMSN’s dynamic network topology, the target sink initiates the data transfer, thereby avoiding searches for active users, untargeted packets, and meaningless retransmissions. In addition, as a measure aimed at the data delay tolerance of DTMSN, EQ-MAC uses dynamic priority queuing management to handle the data packets in the queue. This management strategy is free from the packet blocking constraint.

### 2.1. Neighbor Updating

In DTMSNs, asynchronous duty cycling is preferred over synchronous duty cycling. A synchronous duty cycling does not require nodes to share schedule information and require them only to stay awake long enough to sample the medium. It avoids overheads and excessive energy consumption. The application environment of EQ-MAC is delay tolerant, and data is not necessary to be sent immediately to the sink nodes. So there is no need to guarantee mobile nodes can wake up simultaneously when they are within each other’s communication range. If they are awake, the sink node will send MPF to achieve synchronousness. Otherwise they are waiting for the next appropriate occasion to exchange data and the nodes do not have to be wakened up to maintain synchronicity.

**Figure 1.**
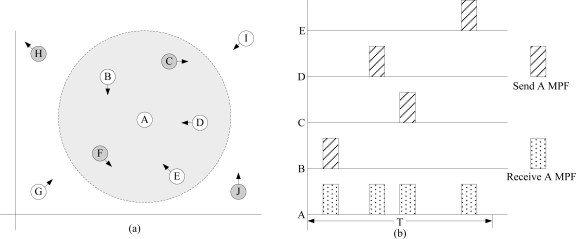
Neighbor Updating.

EQ-MAC employs a preambleless asynchronous scheme. Instead of a long preamble or a series of short preamble packets sent before the data packets, each node will wake up periodically and send MPF. The awake neighborhood nodes will complete the neighbor discovery process once the MPF packets are received. In EQ-MAC’s asynchronous duty cycle, each node broadcasts an MPF containing its own ID on schedule to notify all its neighbors of its identity. Upon receiving the MPF, awake neighbor nodes will update their neighbor tables immediately. As Figure 1(a) shows, nodes move in random directions, as denoted by the arrows. The rotundity field is the communication area of Node A; Nodes B, C, D, E and F can contact Node A, but Nodes G, H, I and J cannot. The dark nodes, *i.e.*, Nodes C, F, H, and J, are asleep during the fixed listening period of *T*. In Figure 1(b), once Node A wakes up, it starts listening to the channel. During the listening period, nodes in the communication range of Node A and that are awake broadcast MPFs. Nodes will detect the channel before sending MPF, and if there is communication in the channel, the nodes will back off for a random time. Otherwise, MPF will be sent immediately. Node A receives MPFs from its neighbor Nodes B, C, D, and E. Note that while Node C is asleep at the beginning of *T*, it wakes up later and broadcasts an MPF on schedule. Node F stays asleep during *T*, so Node A does not receive any information from it. When the listening period is over, Node A immediately updates its neighbor table according to the received MPFs.

### 2.2. Data Delivery Initiated by Target Sink

Upon receiving the MPF, an awake neighbor node will check whether it has data waiting to be sent. If it does not, it will go to sleep. Otherwise, it will parse the MPF, get the ID and determine whether the target sink is the node sending the MPF. The neighbor node as a sender will send a WTS immediately after finding the target receiver, or else it will go to sleep. WTS is broadcasted so that other neighbor nodes will postpone their data transmission to avoid the hidden node problem. After receiving a WTS, the node that sent the MPF will know that there is data waiting for its acceptance. It then broadcasts an RTR as a response to inform the sender node waiting to start data transfers, since the target sink is ready to receive, and to notify the other awake neighbor nodes to go asleep until the whole communication is finished. The communication time is contained in the RTR frame. After three negotiations with the target receiver, the sender begins to transfer valid data.

**Figure 2.**
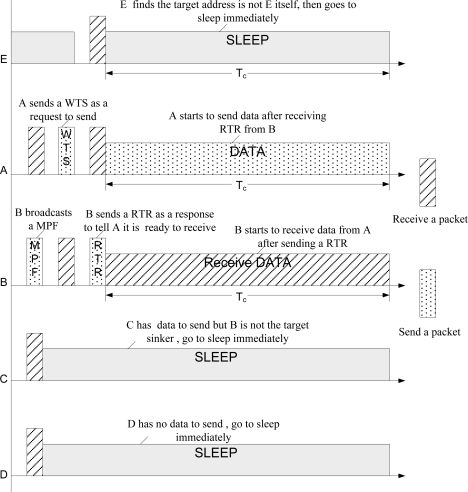
Data Delivery Initiated by Target Sink.

Figure 2 illustrates sensor nodes running EQ-MAC in an asynchronous duty cycle. Node B, when it awakes, broadcasts an MPF to show its existence and inform other nodes of its waking state. The waking neighbor nodes (A, C, D) receive the MPF, get the ID of Node B contained in the MPF, and check to see if each node has data waiting to send. Node D has no data to be sent and thus goes to sleep immediately.

Although there is data waiting for Node C to send, the target sink is not Node B as its ID is not the same. Hence, Node C also goes to sleep. Node A, once it finds it has data waiting to be sent and the target receiver is just Node B, sends a WTS to tell Node B there is data waiting for its acceptance. After receiving the WTS from Node A, Node B sends an RTR as a response to inform Node A about starting the data transfer, which completes the data delivery scheme initiated by the target sink since the first time Node B sent an MPF as a request to receive data packets. RTR is also a message to tell other neighbor nodes to go asleep until the time interval Tc elapses. Like Node E, it is not woken till the interval in which RTR is broadcasted arrives. When receiving the RTR from Node B, Node E gets the time needed to complete the whole communication and the transmission address (TA) in the frame RTR. When the address of Node E does not match the TA, that is, Node B is not ready to receive data from Node E, it goes to sleep until the communication between Node A and Node B is finished. Node A begins to transmit data packets once it receives the RTR. At this point, the three-way handshake initiated by the target sink finishes and valid data transmission starts. By using data delivery initiated by the target receiver, reducing untargeted packet transfers, and avoiding meaningless data retransmissions which happen in highly dynamic network environments, EQ-MAC achieves significant energy saving and assures valid data transfers.

### 2.3. Queue Management Strategy Based on Priority

For the delay-tolerant characteristics of DTMSN, sensor nodes tend to have a data queue that contains data messages ready for transmission. A queue management strategy would appropriately process the data messages in the queue, sort the right data message to be sent when the sensor node meets another, and determine which data message should be dropped when the queue is full. In EQ-MAC, we employ a priority-based queue management scheme.

**Figure 3.**
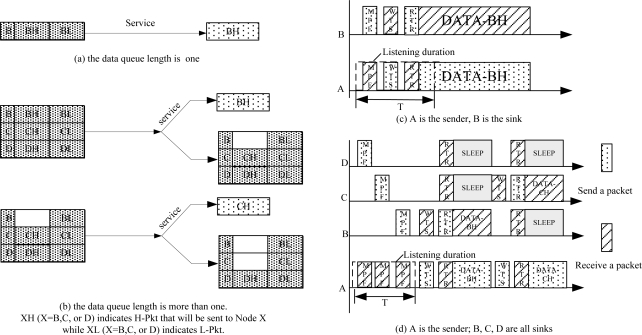
Data Queue.

We distinguish two types of packet by their own priority. H-Pkts have priority over L-Pkts and are serviced first when both packet types are stored in the same data queue. In Figure 3, the number in the panel represents the priority of the data packet. The smaller the number is, the higher the priority the data packet is.

In Figure 3(a), there is only one group of packets in the data queue which would be sent to Node B, and undoubtedly they can be served once the sensor node meets the target sink (Node B) and receives its MPF. The sender will first send H-Pkts (BH as shown in Figure 3(a)) for their higher priority. Figure 3(c) shows the data transmission process between Node A and B. The listening duration will end in advance when Node A meets Node B and detects Node B as the target. Then data transmission starts. When the data queue length is more than one, as shown in Figure 3(b), more than one group of data messages exist in the data queue. The data messages are grouped together according to the ID of their target nodes. Each panel stands for one group of data messages and sorts the H-Pkts and L-Pkts that will be sent to the same sink node according to their own priority. The creation of the data queue and data packets sorting process can be handled efficiently, so the extra cost of the queue management in our MAC protocol implementation is negligible. Once the sensor meets the right receiver it prefers to send the H-Pkts.

For instance, when Node A has data to send, it first detects whether the data queue length is more than one. If it is only one, it will listen to the channel and wait for the MPF of the target sink (Node B). The listening process ends when Node A finds the target and receives an MPF from Node B (Figure 3(c)), and then data transfer starts. When the data queue length is more than one, the sensor needs to choose which data it should send first. As shown in Figure 3(b), there are three groups of data messages existed in the data queue of Node A, which need to be sent to Node B, C, and D. Each group sorts its data as H-Pkt and L-Pkt according to its priority. The data transmission process is shown in Figure 3(d). Node A first listens to the channel for a duration T, looking for the right target (Node B, C, and D) until T elapses. If it finds, it responds with a WTS immediately and starts data communication (between A and B). RTR broadcasts the communication time and other nodes (Node C and D) go to sleep during the duration. After the data transfers finish, the subsequent data transmission starts.

When the data queue is full, the sensor node will determine which group of data messages should be dropped. In EQ-MAC, the drop strategy is based on the priority. The higher priority the data message has, the later they will be dropped by the sensor. After new data has been gathered, the sensor finds there is no more space left for storing the incoming data messages and then compares the priority of the new data with the lowest priority of the messages in the data queue. If the new message has higher priority than the lowest priority in the original queue, the sensor will drop the lowest-priority data messages, push the new data into the data queue instead, prioritize the data messages in the data queue, and reorder them according to their priority. If the new message does not have higher priority, the sensor will drop the new incoming data messages.

## 3. Queuing Analysis

### 3.1. Markov Chain

We developed a discrete-time Markov arrival process (MAP) priority queuing model to analyze the performance of the EQ-MAC protocol in the DTMSN environment. The queuing model is a preemptive priority queuing process [16]. Two conditions are assumed according to the preemptive priority queuing process in our paper. First, a communication preemptive priority queuing process, which means the ongoing communication will be interrupted and the new incoming data group will be preemptive to be sent to the sink if the contact between nodes exists and the incoming data messages have the highest priority; Second, data preemptive priority queuing process, that is to say, the incoming data will be preemptive to join in the data queue according to its priority without affecting the ongoing communication if there is any. When data packets are being sent between the sender and the sink, the priority of the new incoming data messages will be checked. If the incoming data has the higher priority than all the data in the queue including the sending packets and it is identified as the emergency data, the communication will be preempted by the new incoming emergency data. The above is the communication preemptive priority queuing process. While there is no communication between nodes, the incoming data messages will join in the data queue according to its priority. Another situation is that, though the sender is contacting with the sink, the incoming data has the higher priority than all the data left in the queue but the lower (or the same) priority than the sending packets, or it is not emergency enough to stop the ongoing contact, it will also join in the data queue according to its priority and does not affect the ongoing communication. The two preemptive processes are suitable for the different conditions when meeting the incoming data of different priority. This queuing model can deal with the dynamic queue management of DTMSN effectively. Packet arrival is assumed to follow a Poisson process, a simple case of MAP in which *λ*_1_ and *λ*_2_ are the probabilities of arrival for H-Pkts and L-Pkts, respectively. The probability density function [17] is:

(1) $$P_{k}(t)=\;\lambda_{k}e^{-\lambda_{k}t}\;\;\;\;(t\;\geq \;0\;,\;\;\;k\;=\;1\;\text{or}\;2\;)$$

The service time (*i.e.*, the time elapsed when sending the data packet, which is proportional to the length of the packet) distributions of the packets follow an i.i.d. negative exponential distribution, where *μ*_1_ and *μ*_2_ are the service rates for H-Pkts and L-Pkts, respectively:

(2) $$P_{k}(s)=\;\mu_{k}e^{-\mu_{k}s}\;\;\;(\;s\;\geq \;0\;,\;\;\;k\;=\;1\;\text{or}\;2\;)$$

The number of H-Pkts in the data queue is i, and the number of L-Pkts is j. Let *p*(*i*, *j; t*) is the probability of i H-Pkts and j L-Pkts in the data queue at t.

The system utilization factor is:

(3) $$\rho_{k}\;=\;\frac{\lambda_{k}}{\mu_{k}}\;\;(k\;=\;1\;\text{or}\;2)$$

Given $\rho_{1}+\rho_{2}\;=\;\frac{\lambda_{1}}{\mu_{1}}\;+\;\frac{\lambda_{2}}{\mu_{2}}\;<\;1$, the balance equation is:

(4) $$\left\{ \begin{array}{l} \left(\lambda_{1}+\lambda_{2}\right)\;p(0,0)\;=\;\mu_{1}\;p(1,0)\;+\;\mu_{2}\;p(0,1)\;,\\ \left(\lambda_{1}+\lambda_{2}+\mu_{1}\right)\;p(i,0)\;=\;\lambda_{1}\;p(i-1,0)\;+\;\mu_{1}\;p(i+1,0)\;,\;i>0\;,\;j=0\\ \left(\lambda_{1}+\lambda_{2}+\mu_{2}\right)\;p(0,\;j)\;=\;\lambda_{2}\;p(0,\;j-1)\;+\;\mu_{1}\;p(1,\;j)\;+\;\mu_{2}\;p(0,\;j+1)\;,\;i=0\;,\;j>0\;,\\ \left(\lambda_{1}+\lambda_{2}+\mu_{1}\right)\;p(i,\;j)\;=\;\lambda_{1}\;p(i-1,\;j)\;+\;\lambda_{2}\;p(i,\;j-1)\;+\;\mu_{1}\;p(i+1,\;j)\;,\;i>0\;,\;j>0\;, \end{array}\right.$$

where $p(i,j)\;=\underset{t\to \infty }{\text{lim}}\;p(i,j;t)\;(i,j\geq 0)$.

To solve (4), we introduce the generating function:

(5) $$\psi_{i}\;(z)\;=\;\sum_{j=0}^{\infty }p(i,j)z^{j}\;,\;|z|\;<\;1\;,\;i\geq 0$$

(6) $$\psi (u,z)\;=\;\sum_{i=0}^{\infty }\psi_{i}\;(z)u^{i}=\sum_{i=0}^{\infty }\;\sum_{j=0}^{\infty }p(i,j)u^{i}z^{j}\;(|u|\;<\;1\;,\;|z|\;<\;1)$$

The state probability is:

(7) $$p(i,j)\;=\;\frac{1}{i!j!}{\left[\frac{\partial^{i+j}\psi (u,z)}{\partial u^{i}\partial z^{j}}\right]}_{i=j=0}$$

Let z → 1 and *p_i_* be the probability when the number of H-Pkts is i. We get:

(8) $$\;p_{i}\;=\;\left(1-\rho_{1}\right)\rho_{1}^{i}\;,\;i\geq 0$$

Let *u* → 1 and *p_j_* be the probability when the number of L- Pkts is *j*. We get:

(9) $$\sum_{j=0}^{\infty }jp_{j}\;=\;\frac{\rho_{2}}{\left(1-\rho_{1}-\rho_{2}\right)}\left[1+\frac{\mu_{2}\rho_{1}}{\mu_{1}(1-\rho_{1})}\right]$$

From Equation (8), we can easily obtain the average number of H-Pkts:

(10) $$E\left[N_{1}\right]\;=\;\frac{\rho_{1}}{1-\rho_{1}}$$

From Equations (9) and (10), we get the average number of L-Pkts:

(11) $$E\left[N_{2}\right]\;=\;\frac{\rho_{2}}{1-\rho_{1}-\rho_{2}}\;\left[1+\frac{\mu_{2}}{\mu_{1}}E\left[N_{1}\right]\right]$$

### 3.2. Performance Measures

#### 3.2.1. Mean Packet Delay

The mean packet delay *D* is the average delay from the time data is sampled and queued to the time the packet is sent successfully. It can be obtained from Little’s Law [17], as follows:

(12) $$D\;=\;\frac{N}{\lambda }$$

where N is the average number of packets in the buffer of the sensor, and *λ* is the average probability of arrival for data packets.

From Equations (10), (11) and (12), we get:

(13) $$D\;=\;\frac{E\left[N_{1}\right]\;+\;E\left[N_{2}\right]}{\lambda_{1}+\lambda_{2}}\;=\;\frac{1}{\left(\lambda_{1}+\lambda_{2}\right)}\left[\frac{\rho_{2}}{1-\rho_{1}-\rho_{2}}\left[1+\frac{\mu_{2}}{\mu_{1}}\times \frac{\rho_{1}}{1-\rho_{1}}\right]+\frac{\rho_{1}}{1-\rho_{1}}\right]$$

#### 3.2.2. Packet Drop Probability

The packet drop probability can be found by using the steady probability for the queue length of N_1_ H-Pkts (8), as follows:

(14) $$p_{d}\;=\;\lambda_{1}^{-1}\times \left(1-\frac{\rho_{1}}{1+\rho_{1}}\right)\times \left(1-p_{N_{1}.}\right)=\;\frac{1}{\lambda_{1}\left(1+\rho_{1}\right)}\times \left[1-(1-\rho_{1})\rho_{1}^{E\left[N_{1}\right]}\right]$$

where *E*[*N_1_*] is obtained from (10).

#### 3.2.3. Throughput

The system throughput [18] can be obtained from Equation (14):

(15) $$S=\;\lambda_{1}\;\times \;\left(1-p_{d}\right)\;=\;\lambda_{1}\;\times \;\left\{1-\frac{1}{\lambda_{1}\left(1+\rho_{1}\right)}\times \left[1-\left(1-\rho_{1}\right)\rho_{1}^{E[N_{1}]}\right]\right\}$$

## 4. Optimization

We shall use an efficient mean estimation method to estimate the parameters related to EQ-MAC; that is, the mean value will be used to estimate the various values needed. The objective function is:

(16) $$\varphi \left(t_{i},\;\;t_{r},\;\;t_{l}\right)=\;\varphi \left({\hat{t}}_{i},\;{\hat{t}}_{r},\;{\hat{t}}_{\iota }\right)=\;\varphi \left(E\left[t_{i}\right],E\left[t_{r}\right],E\left[\;t_{l}\right]\right)$$

Here, *t_i_* is the listening time from the moment one node is woken to the time it receives an MPF, *t_r_* is a randomly chosen back-off time after receiving the MPF, and *t_l_* is the listening time after the sink sends an MPF and before it receives the WTS. ${\hat{t}}_{\iota }$, ${\hat{t}}_{r}$ and ${\hat{t}}_{l}$ are the estimated values of *t_i_*, *t_r_*, and *t_l_*. E[*t_i_*], E[*t_r_*], and E[*t_l_*] are the mean values of *t_i_*, *t_r_*, and *t_l_*.

We denote the average number that nodes contact in 1 second to be n and the mean number of data exchanges in each contact to be P. The time for checking the idle channel is *t_c_*. *L_p_* is the data packet length for one contact. *L_M_*, *L_W_*, and *L_R_* are the control packet lengths of MPF, WTS, and RTR. *C_r_*, *C_t_*, and *C_s_* are the average consumed current when nodes are receiving packets, transmitting packets, and asleep. The data rate is *r_d_* and the supply voltage is *V*.

The average energy consumed by nodes sending packets in 1 second is:

(17) $$\begin{array}{lll} E_{t} & = & \text{energy}\;\text{for}\;\text{idle}\;\text{listening}\;+\;\text{energy}\;\text{for}\;\text{listening}\;\text{during}\;\text{back-off}\;\text{time}\\ & & +\;\text{energy}\;\text{for}\;\text{receiving}\;\text{MPF}\;\text{and}\;\text{RTR}\;+\;\text{energy}\;\text{for}\;\text{sending}\;\text{data}\;+\;\text{energy}\;\text{for}\;\text{sending}\;\text{WTS}\\ & = & \mathrm{nV}\left[\left(E\left[t_{i}\right]+E\left[t_{r}\right]+\frac{P\left(L_{M}+L_{R}\right)}{L_{p}r_{d}}\right)c_{r}+\left(\frac{P}{r_{d}}+\frac{PL_{W}}{L_{p}r_{d}}\right)c_{t}\right] \end{array}$$

The average energy consumed by nodes receiving packets in 1 second is:

(18) $$\begin{array}{lll} E_{r} & = & \text{energy}\;\text{for}\;\text{detecting}\;\text{the}\;\text{channel}\;+\;\text{energy}\;\text{for}\;\text{listening}\;\text{after}\;\text{sending}\;\text{MPF}\;\text{and}\;\text{before}\;\\ & & \text{receiving}\;\text{WTS}\;+\;\text{energy}\;\text{for}\;\text{receiving}\;\text{data}\;+\;\text{energy}\;\text{for}\;\text{receiving}\;\text{WTS}\\ & & +\;\text{energy}\;\text{for}\;\text{sending}\;\text{MPF}\;+\;\text{energy}\;\text{for}\;\text{sending}\;\text{RTR}\\ & = & \mathrm{nV}\left[\left(t_{c}+E\left[t_{l}\right]+\frac{P}{r_{d}}+\frac{PL_{W}}{L_{p}r_{d}}\right)c_{r}+\left(\frac{L_{M}+L_{R}}{r_{d}}\right)c_{t}\right] \end{array}$$

The average energy consumption when nodes are idle in 1 second is:

(19) $$\begin{array}{lll} E_{i} & = & (\text{idle}\;\text{listening}\;\text{times - listening}\;\text{times}\;\text{with}\;\text{data}\;\text{exchange})\times (\text{energy}\;\text{for}\;\text{channel}\;\text{detecting}\;+\\ & & \text{energy}\;\text{for}\;\text{channel}\;\text{listening}\;+\;\text{energy}\;\text{consumed}\;\text{by}\;\text{sending}\;\text{MPF}\;\text{for}\;\text{neighbour}\;\text{updating})\\ & = & \left(\frac{1}{E\left[t_{i}\right]}-n\right)V\left[t_{c}+E\left[t_{l}\right]c_{r}+\frac{L_{M}c_{t}}{r_{d}}\right] \end{array}$$

The average energy consumption when nodes are asleep in 1 second is:

(20) $$\begin{array}{ll} E_{s} & =\text{energy}\;\text{comsumption}\;\text{during}\;\text{sleeping}\\ & =V\{1-n\left[E\left[t_{i}\right]+E\left[t_{r}\right]+\frac{P}{r_{d}}\left(1+\frac{L_{M}+L_{W}+L_{R}}{L_{p}}\right)\right]-n\left[t_{c}+E\left[t_{l}\right]+\frac{P}{r_{d}}\left(1+\frac{L_{W}}{L_{p}}\right)\;+\frac{L_{M}+L_{R}}{r_{d}}\right]\\ & -\left(\frac{1}{E\left[t_{i}\right]}-n\right)\left(t_{c}+E\left[t_{l}\right]+\frac{L_{M}}{r_{d}}\right)\}c_{s} \end{array}$$

The total average energy consumption of the network in 1 second is:

(21) $$E=E_{t}+E_{r}+E_{i}+E_{s}$$

For energy efficiency, *E* should be minimized. We simply assume the mean value of the time is half of the time window and maximize the network lifetime by adjusting *t_i_*, *t_r_*, and *t_l_*:

(22) $${\hat{t}}_{x}=\;E\left[t_{x}\right]=\frac{t_{x}}{2}\;(x=i,r,l)$$

From Equations (17–22), we get:

(23) $$\frac{\partial E}{\partial t_{i}}=\frac{1}{2}\mathrm{nV}\left(C_{r}-C_{s}\right)-\frac{1}{t_{i}^{2}}V\left\{\left(t_{c}+t_{l}\right)\;\left(C_{r}-C_{s}\right)+\frac{L_{M}}{r_{d}}\left(C_{t}-\;C_{s}\right)\right\}$$

To minimize *E*, let $\frac{\partial E}{\partial t_{i}}=0$. Accordingly, we get:

(24) $$t_{i}=\sqrt{\frac{2}{n}\left[t_{c}+t_{l}+\frac{L_{M}\left(c_{t}-c_{s}\right)}{r_{d}\left(c_{r}-c_{s}\right)}\right]}$$

*t_l_* can be chosen according to empirical values and series of experimental results, thus *t_i_* can be obtained from (24). By repeating the above process, groups of (*t_l_*, *t_i_*) can be recoded. And the best parameters can be fixed from the further experiments. Then *E* can be approximately minimized with the best values between *t_i_* and *t_l_*. Via the mean estimation method and optimized adjustment of experimental parameters, we can set up the network model and carry out successive experiments.

## 5. Performance Evaluation

### 5.1. Experimental Setup

**Figure 4.**
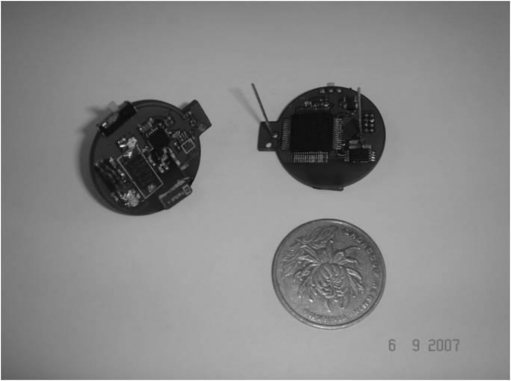
ISC_Mote.

To evaluate EQ-MAC, we performed a series of simple experiments on our experimental platform using ISC_Motes, as shown in Figure 4.

The radio used by ISC_Mote is the NRF24L01 chip, which has a maximum data rate of 2 Mbps and operates in the 2.4-GHz ISM band. The mote uses a TI MSP430F149 processor and fixes the temperature sensor DS620 to measure temperature. The experiment applications are based on the tinyOS [19], which is an open source operating system, designed by UC Berkeley for WSNs. In EQ-MAC, nodes sleep in power-down mode. Moreover, they are in fact in Standby-I mode when they are idle listening [20].

We chose a playground about 10,000 m^2^ in our campus as the experiment field. The communication distance of the mote is about 10 m. Twenty members of our institute were invited to join in the experiment. Each wore a sensor tag. The sensors sampled their carrier’s human body temperature by DS620 while the participants moved around. Each participant could move randomly, which means he could move around within the playground or out of it as he wanted. The mote sampled the temperature every second, and stored the data into the queue. Data packets were grouped according to the ID of their sink, while each packet was tagged for a priority in every group. H-Pkts were generated by some of the participants who took strenuous exercise before the experiment and those whose temperature were slightly higher for the individual differences. If the average temperature was higher than 37.8 °C, the data packet was handled as H-Pkt, otherwise it was treated as L-Pkt. According to our experiment, the H-Pkts were about 25% in all the packets. When the sink was in the communication range of the sender and the handshake process was finished, packets would be sent to the sink. This experimental scene met with the network environment of DTMSN, where nodes were mobile, and data packets were delay-tolerant and could be gathered for a period, waiting for the suitable moment to be sent. We made a series of experiments by changing the node density and the packet arrival rate with each experiment for 3 hours. The experimental parameters are listed in Table 1.

### 5.2. Numerical Results

We implemented three MAC protocols in the experiment, EQ-MAC, nQ-MAC, and AS-MAC. EQ-MAC is the protocol with the dynamic queue management strategy as mentioned above. NQ-MAC follows EQ-MAC’s receiver-initiated data transmission scheme but with no data priority, and the queue management without priority is used, thus the queuing model is the traditional queuing process. The performance analysis can be easily obtained like the derivation in Section 3. AS-MAC is the protocol proposed in [12]. Numerical results are presented to show that EQ-MAC with receiver-initiated data transmission scheme outperforms AS-MAC which is commonly used in the traditional wireless sensor network in energy efficiency. The results also show that with the same receiver-initiated transmission scheme, EQ-MAC employing the dynamic queue management strategy can improve the probability of success in data transmission. The service rate of H-Pkt *μ*_1_was fixed at 0.9, while the service rate of L-Pkt *μ*_2_ was set at 0.6.

#### 5.2.1. Energy Efficiency

**Figure 5.**
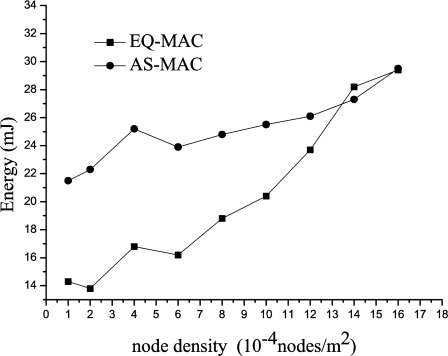
Energy Consumption.

In a delay-tolerant and mobile network environment with a highly dynamic and sparse network topology, the average energy consumption in EQ-MAC visibly decreases compared with other MAC protocols in which the sender always initiates data transfers first. As Figure 5 shows, when the nodal density is less than 10, EQ-MAC yields a more efficient power saving in comparison with AS-MAC. The reason for this difference is their different data transmission mechanisms. In conventional MAC protocols like AS-MAC, data transfers are initiated by the sender. In a mobile and sparse network environment, this can easily lead to a transmission failure because the target node may be not in the contact area of the sender, and the consequently useless retransmission would increase energy consumption. But in EQ-MAC, it is the intended sink that initiates efficient data transfers through the neighbor updating process. This scheme assures successful data transmission, reduces the probability of data retransmission, and decreases transmissions of control packets; the result is higher energy efficiency.

#### 5.2.2. Mean Packet Delay

**Figure 6.**
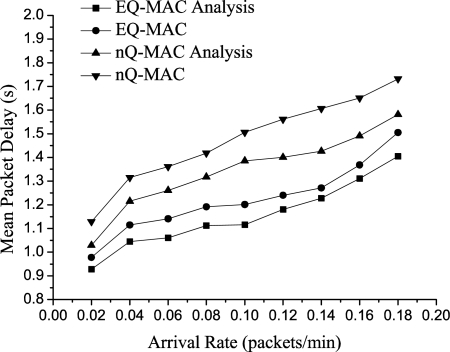
Mean Packet Delay *vs*. Arrival Rate.

Figure 6 plots the mean packet delay. The mean packet delay for EQ-MAC is 25% less than that of the conventional MAC protocol. Such a significant difference in favor of EQ-MAC is attributed to the dynamic queue-management scheme based on priority. In the conventional protocol, packets are transmitted according to their arrival sequence. On the other hand, the transmission sequence in EQ-MAC is based on the priority of packets, and we can dynamically manage the data queue. Thus, useful information can arrive in a more timely manner than in the non-queue-management strategy. Note that in the experiments, data packets were stored in memory and were sent or received by the hardware components of the ISC_Mote. There were thus delays in the communications between the hardware components. This means the analytical results always appear to be better than the experimental ones.

#### 5.2.3. Packet Drop Probability

**Figure 7.**
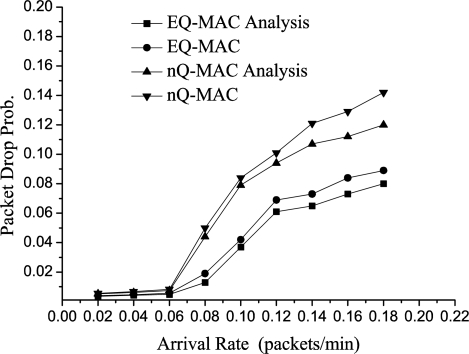
Packet Drop Prob. for H-Pkts *vs*. Arrival Rate.

Figure 7 plots the packet drop probability for H-Pkts against the effective arrival rate. We can see that the packet drop probability for H-Pkts in EQ-MAC becomes lower than that of the non-queue-management strategy as the arrival rate increases.

When the arrival rate is 0.12, EQ-MAC has a 46% decrease in packet loss compared with the non-queue-management strategy. Packet loss decreases since the dynamic queue-management scheme based on priority enables effective and rapid data processing and decreases blocked packets and accumulations of packets. Differences between theoretical analysis and experiments occur, for instance, when error bits occur during data transmission; the hardware components check the CRC, and when they find an error, they discard the packet. This is not accounted for in the theoretical analysis. Thus, there are always differences between the results of the analysis and the experiments.

#### 5.2.4. Throughput

**Figure 8.**
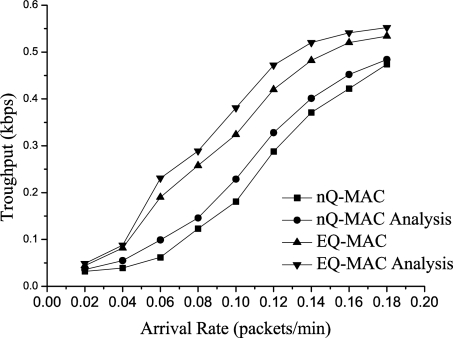
Throughput for H-Pkts *vs*. Arrival Rate.

**Figure 9.**
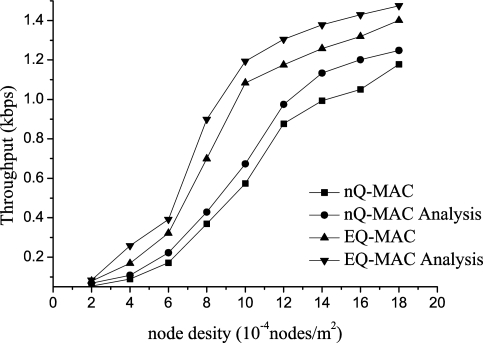
Throughput for H-Pkts *vs*. Node Density.

Figures 8 and 9 show the throughput for H-Pkts. When the network topology is sparse, EQ-MAC has better throughput because the process of data transfers initiated by the target sink ensures successful data transfers.

Furthermore, the dynamic queue-management strategy increases the efficiency of H-Pkt transmission. When the node density is 10, the system throughput increases by 88.9% in comparison with the non-queue-management MAC protocol. When the arrival rate is 0.10, EQ-MAC has 79% increase in throughput compared with the non-queue-management MAC protocol. The results of the experiments and the theoretical analysis were somewhat different because the sending and receiving processes of the hardware were different from those of the theoretical analysis. For example, when data packets appear with high frequency, the wireless hardware module for sending and receiving data would have not enough time to deal with them or sufficient memory to accept and store some of them. Hence, some of these packets were discarded and could not be received in the experiment.

## 6. Conclusions

Nodal mobility and delay-tolerant data are two key characteristics in DTMSNs. Typically in medical treatment such as the epidemic tracking, infectious diseases are isolated and each wears a sensor tag for epidemic control. In this case nodes are mobile, and most sampling data is gathered for statistical analysis and only some urgent data is required to be sent immediately after it is sampled. In athletes’ training surveillance, each athlete catches a sensor node to sample his body information like body temperature and heartbeat, which provides data for the coach to analyze and this information can be used to improve the athletes’ training effort. These special mobile scenes with low node density and delay-tolerant data meet the application of DTMSN, and require an effective medium access control scheme to solve the problems of energy saving and data queue management.

Numerous schemes like duty cycling have been proposed for the medium access control layer of WSNs to avoid idle listening and reduce energy consumption. These schemes, however, operate poorly or do not have enough availability to work in the delay-tolerant mobile network environment of DTMSN without excessive energy consumption and data packet losses. We have proposed an energy-efficient MAC protocol using dynamic queue management called EQ-MAC as a way of dealing with the special characteristics of a highly dynamic and sparse topology and poor network connectivity in DTMSNs. Via data delivery initiated by the targeted sink, EQ-MAC guarantees that target gets the data intended for it, and it increases the data transfer rate and consumes less power in comparison with other protocols. Moreover, the targeted data delivery scheme reduces ineffective data packet retransmissions and useless transfers of control packets, leading to another obvious energy saving. EQ-MAC uses a dynamic queue management strategy based on priority to decrease the packet drop probability and mean packet delay. The strategy makes useful data reach the target terminal in a more timely manner. Experiments on a sensor platform verified that the dynamic strategy of EQ-MAC outperforms traditional MAC protocols not only in terms of energy consumption and mean packet delay but also in terms of packet drop probability and throughput in a DTMSN environment with highly dynamic and sparse topology.

## Figures and Tables

**Table 1.** Experimental Parameters.

***Parameter***	***Value***
Transmission Current (mA)	23.4
Receive Current (mA)	25.8
Listen Current (uA)	148
Sleep Current (nA)	900
Supply Voltage (V)	3.0
Data Rate (kbps)	2,000
Battery Capacity (mAh)	580
Time for check the idle channel (ms)	50
Interval for sending MPF (ms)	500
Sleep period (ms)	500
Listening window (ms)	100
Length of MPF (bytes)	5
Length of RTR (bytes)	10
